# Self-Assured and Sober: The Relationship Between Maternal Parenting Sense of Competence, Stress, and Alcohol Use

**DOI:** 10.3389/fgwh.2021.778183

**Published:** 2022-01-31

**Authors:** Erin Johnson, Rebecca Fellowes, Kelsie Cant, Sally Hunt

**Affiliations:** School of Psychological Sciences, The University of Newcastle, Callaghan, NSW, Australia

**Keywords:** parenting, alcohol, stress, competence, mother

## Abstract

Alcohol misuse is widespread, creating serious health and parenting harms. It is important to explore the motivations behind why people drink and the modifiable factors determining severity of the behavior. While alcohol-related research has historically focused on men, the closing gender gap in alcohol consumption highlights a need for targeted research on women. Parenting stress is a commonly reported motivation for maternal drinking. Likewise, parenting stress is associated with parenting sense of competence. However, there is no research connecting parenting sense of competence with alcohol use directly, nor indirectly via moderation of the alcohol and parenting stress relationship. The current study explored these associations and investigated the potential moderation through a questionnaire completed by a sample of 406 mothers. There were significant correlations between all factors, however, parenting sense of competence was not a significant moderator of the parenting stress and alcohol use relationship. Specifically, as a mother's parenting stress increases, her confidence in the parenting role tends to decline and she is more likely to misuse alcohol. Despite this, variation in parenting sense of competence among women was not significantly correlated with one's likelihood to drink when coping with stress. Further exploration of these relationships is required, with replication of the current study following the COVID-19 pandemic.

## Introduction

In Australia, alcohol has long been viewed as a symbol of mateship and camaraderie, particularly among males, forming part of the romantic Australian legend (1, 2). Despite being the most commonly treated substance use problem in Australia (3), alcohol is the only substance where approval of regular use by adults (45%) is higher than disapproval [21%; (4)]. According to the AIHWs National Drug Strategy Household Survey 2019 (4), ~35% of the population (aged 14 years and over) consume alcohol at least once per week, with 5% drinking daily. However, in recent decades, a growing research base has highlighted the damaging effects of alcohol use, with a strong focus on the health consequences and motivations behind such behavior. Alcohol is a substance which depresses the central nervous system, affecting neurotransmission and autonomic activity (5). It inhibits motor and sensory processes, slowing cognition and impeding normal judgement (6). The use of alcohol is motivated by four major factors—social, enhancement, conformity and coping (7), the most concerning of which is coping (8). One major trigger for the need to cope is stress (9), with parenting stress forming one facet of this construct (10). Parenting stress is defined as the stress experienced in relation to the role of being a parent, as influenced by child, parental or situational characteristics (11), and is a commonly reported reason for alcohol consumption (12). While a substantial proportion of research literature has found that parenting stress is positively correlated with maternal alcohol consumption (12), some studies have found the opposite (13). One possible explanation is that one's parenting sense of competence moderates the relationship between parenting stress and alcohol consumption. Parenting sense of competence is similar to the construct of self-efficacy, referring to one's self-referent estimations of parenting ability (14). Investigating the factors behind alcohol consumption could identify new target areas for intervention in alcohol misuse, working to prevent adverse health and social consequences. Therefore, the current study will use a parenting competence-based explanation to understand mothers' drinking to cope.

Traditionally, males have made up the larger portion of alcohol consumers, with 85% of males consuming alcohol compared with 79% of females in 2019 (15). However, studies worldwide have indicated increasing gender convergence in alcohol consumption throughout recent decades (16–18). This narrowing of the gap is more rapid in countries abandoning traditional gender roles, where there is greater opportunity in employment, education and access to reproductive health services (19). The exposure to new sources of interpersonal stress due to the juggling of career and caregiving may facilitate the use of alcohol as a coping mechanism (20). It is important to note that this trend involves many complex factors, including the hastening of convergence due to a general reduction of drinking by males, and the stronger driving forces of particular female age groups (16, 17). Specifically, data from 2018 shows that 11% of women aged 35 to 44 exceeded the lifetime risk guideline [more than two drinks on any day; (21)], compared with just 6.1% of those aged 18-24 (22). As 36% of Australian women give birth between the ages of 30 and 34 (23), it is reasonable to assume that the rise in alcohol consumption is influenced by the stresses of early child-rearing.

In addition to these factors, it is evident that there are multiple biological and psychosocial differences between males and females which influence the occurrence, symptoms, comorbidity and treatment of substance use disorders; therefore, research must be separately undertaken for both genders. Despite this, research has been mostly focused on male-only samples (24) highlighting a particular need for research focused on females.

Compared to men, women are much quicker to develop alcohol-related cirrhosis of the liver (25), have more significant increases in risk for heart disease (26), and a 10-30% increase in the risk of breast cancer at just moderate amounts of drinking (27–29). This risk is primarily because when consuming the same proportion of alcohol per kilogram of body weight, a woman's blood ethanol level is higher than a man's (30).

While these consequences are concerning for any individual, women also carry the additional risk of alcohol misuse during pregnancy, with fetal alcohol syndrome having an estimated prevalence of 14.6 in 10,000, and 77.3 in 10,000 people for fetal alcohol spectrum disorders (31). Therefore, maternal drinking, when compared to paternal drinking, is particularly problematic given that it has the potential to directly harm an involuntary third party in addition to the voluntary drinker.

Given these alarming statistics, it is imperative to understand the mechanics behind such behavior. According to Smorti and Guarnieri (7), those who drink to cope are avoiding those emotions that provide internal, negative reinforcement. Of the four alcohol-use motives, coping has been most strongly associated with hazardous alcohol use and alcohol-related problems (8).

To understand coping mechanisms, the “tension reduction hypothesis” theorized that people consume alcohol with the expectation that it will reduce their stress (32). Stress is a broad term used to represent the situations where environmental demand outweighs an individual's perceived psychological and physiological ability to cope effectively (33). The tension reduction hypothesis was recently supported by data collected during April 2020, amid the COVID-19 pandemic, in which 22.8% of women increased their alcohol consumption. The most commonly self-reported reason for this, besides spending more time at home, was increased stress (34). While there are several types of stress, one major type is parenting stress, the accumulation of which can diminish one's ability to manage emotions and eventually drains physical and mental resources, reducing one's capacity to be an effective parent (35, 36), justifying why many parents search for coping strategies.

While alcohol can reduce the immediate perception of stress by dampening senses (37), it has been shown to deteriorate family functioning, exacerbating child behavioral and emotional problems (38). Parental alcohol misuse is correlated with inconsistent discipline (39), reduced monitoring (40), as well as reduced quality of the parent-child relationship, often involving conflict (41). In severe cases, alcohol misuse can hamper parenting ability so heavily that children become victims of abuse and neglect (42, 43). In addition to these abilities, intoxication may make it difficult for parents to complete basic household tasks such as preparing nutritious meals, getting children to school or enforcing regular routines (44). Therefore, it may actually act to worsen stress in the long-term (10, 45). Despite these negative consequences, 43.5% of parents with dependent children under 14 years old were risky drinkers in 2016 (46).

Identifying and understanding modifiable predictors of alcohol use is especially important given the significant impact on children's life outcomes. Specifically, parental alcohol use has been consistently associated with adolescent alcohol consumption (47–49), poorer academic outcomes (50), increased risk of drug use (51), and conduct problems (52) leading to criminal behavior (53). Children's mental health correlates with parental alcohol use, with misuse increasing the likelihood of depression, poor self-esteem and feelings of loneliness, even into adulthood (54, 55).

While this damage can be done by both genders, alcohol misuse seems to be more problematic for mothers than fathers. Although fathers are playing an increasingly larger role in family duties (56), women are thought to still hold primary responsibility for parenting (42). The style of maternal parenting is also indicated to have a greater impact on a child's development than that of the father (57, 58). Additionally, studies by Pelchat et al. (59, 60) reported that mothers are more commonly affected by parenting stresses than fathers. Mothers are also more likely to consume alcohol to regulate these negative emotions and stress in general, while fathers are more likely to drink for positive reinforcement such as stimulation (61). These differences highlight a need to understand the unique factors impacting women, specific to the parenting role.

While the tension reduction hypothesis (62) has been frequently supported by indirect factors such as drinker expectations and motives (63, 64), inconsistent results have emerged when attempting to analyze the theory directly (65). Pelham et al. (12) noted that while many mothers with high parenting stress increased their drinking, a substantial number reduced their consumption. This inconsistency is evident throughout the literature. For example, Breslin et al. (13) found a negative correlation between stress and alcohol consumption, noting that significantly less alcohol was consumed on weeks where stress was increased. This variation suggests the presence of other factors which may moderate the strength or direction of the stress and alcohol relationship.

One potential moderator could be parenting sense of competence, consisting of three factors—parenting satisfaction, self-efficacy and interest (66). Self-efficacy is defined as one's perceived confidence, familiarity and problem-solving ability in the role of a parent (67). While the satisfaction sub-scale is the social value of the role, considering anxieties, motivations and frustrations (68). Interest is simply one's interest in their role as a parent (66). Parenting sense of competence is believed to influence parental behaviors, with differences playing a key role in understanding variances in parenting strategy (69). A study by Jones and Prinz (70) also noted that parenting sense of competence is strongly correlated with one's actual parenting competence.

Several studies have shown inverse correlations between a mother's parenting stress and parenting sense of competence (36, 71, 72) indicating that mothers experience lower parenting stress when they view their parenting abilities more positively. This relationship is found for all factors of parenting sense of competence, but more strongly for parenting satisfaction (71). Interestingly, various studies have noted that fathers are more satisfied in their parenting role than mothers (66, 68, 73). Therefore, given the large weighting of satisfaction in the association with parenting stress, the idea that fathers are less impacted by parenting stresses is supported. It also suggests that targeting a mother's parenting sense of competence, especially focusing on satisfaction, may be one critical strategy in the treatment of maternal alcohol misuse.

Parenting sense of competence potentially influences the implementation of various coping mechanisms, and impacts their perception of how effective and appropriate the behavior is (74). As alcohol is commonly used to cope with stress (64), it is, therefore, plausible that improvements in parenting sense of competence could allow mothers to correctly observe the negative consequences of this method, encouraging them to replace it with more effective mechanisms. This is supported by research showing that a ‘strengths-based' model of intervention is particularly helpful in behavioral health intervention where maladaptive coping strategies are targeted (75, 76). This method helps the client recognize their strengths and build confidence in their abilities, increasing the likelihood of over-coming future stressors (75).

If supported, lower parenting sense of competence may reduce a mother's ability to cope with parenting stress, resulting in increased alcohol consumption, potentially sparking a self-propagating cycle. As alcohol misuse is known to reduce parenting ability, and actual competence is strongly correlated with parenting sense of competence (70), it may be assumed that alcohol consumption further reduces parenting sense of competence, worsening the situation rather than relieving it. Therefore, modifying parenting sense of competence may be key in breaking this cycle, consequently preventing and treating maternal alcohol misuse.

While the connection between parenting stress and parenting sense of competence has been studied, we are not aware of any published studies that have investigated the potential moderating effect of parenting sense of competence on the link between alcohol consumption and parenting stress. We are also not aware of any studies exploring the relationship between alcohol consumption and parenting sense of competence directly. Therefore, the current study first aims to replicate previous literature by reassessing the relationships of parental stress with alcohol and parenting sense of competence. Secondly, it aims to extend upon this research by investigating whether parenting sense of competence is related to alcohol consumption directly, and whether it moderates the relationship between parenting stress and alcohol consumption.

In fulfilling these aims, it is first hypothesized that parenting stress will be positively correlated with alcohol consumption and negatively correlated with parenting sense of competence and, secondly, that there will be a significant negative relationship between parenting sense of competence and alcohol consumption. It is finally hypothesized that parenting sense of competence will moderate the relationship between parental stress and alcohol consumption.

## Materials and Methods

### Design

This study used a quantitative methodology with a non-experimental, cross-sectional questionnaire design to determine non-causal relationships between variables. The independent variable was parenting stress score and the dependent variable was alcohol consumption. The moderator variable was parenting sense of competence.

Based on the literature, relevant covariates were also included for robustness checking. The first covariate was maternal age which was included due to the differing contribution of certain age brackets to the closing gender gap in alcohol consumption (16, 17). Number and age of dependent children were also included given their positive and negative correlations with parenting stress, respectively (77).

### Participants

Participants responded to online advertisements for the study and were eligible if they identified as female, aged 18 years or over, resided in Australia and had at least one dependent child under 18 years of age. While we acknowledge that younger women can be mothers, only those over the Australian legal drinking age were recruited. Only respondents who completed the relevant survey questions were include in the current analysis.

**Figure 1:**
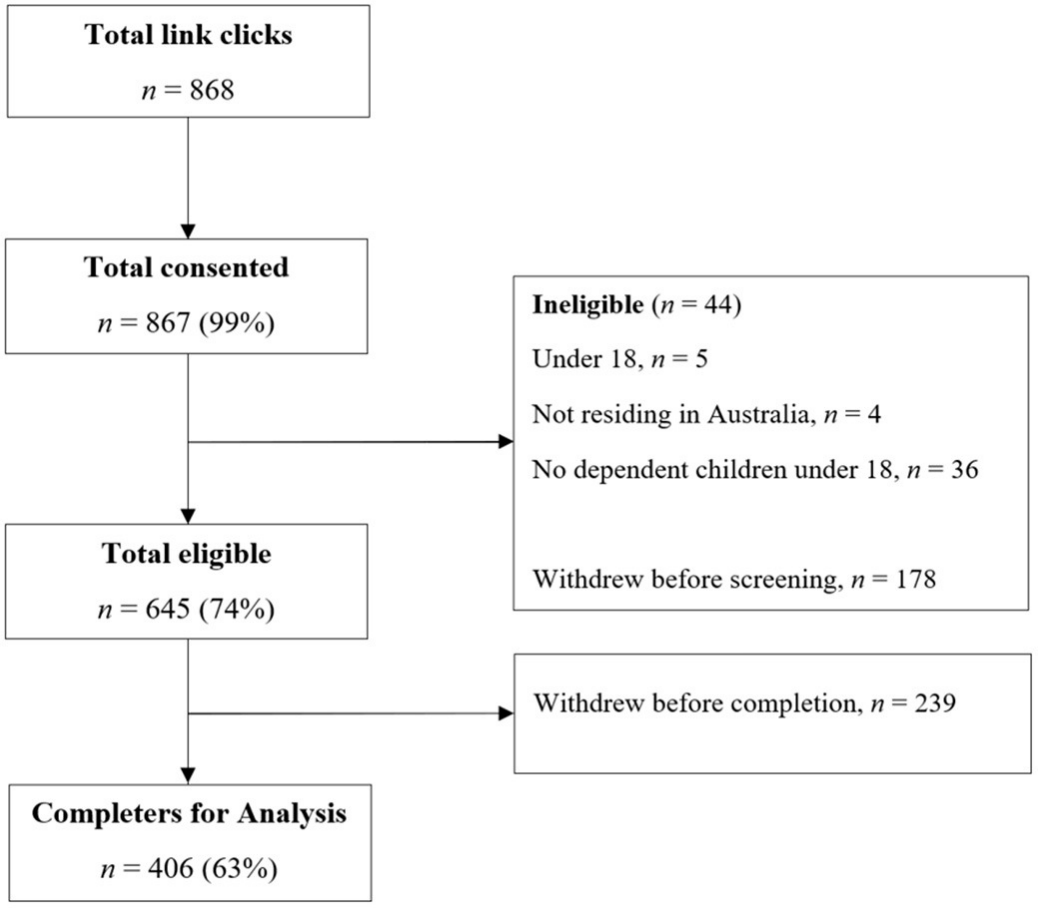
Flowchart of participant recruitment.

Of those who responded to the advertisement, 868 proceeded to the Participant Information Statement, followed by a declaration of consent. Those who consented (*n* = 867) were then screened for eligibility, where 20.5% of consenters voluntarily exited the questionnaire (*n* = 178) and 5.1% were deemed ineligible which ended the questionnaire immediately (*n* = 44). Of the eligible participants (*n* = 645), several ceased responding before completion of the questionnaire (*n* = 239), leaving 406 participants for inclusion in the analysis. The data collection process is summarized in Figure 1.

### Measures

The questionnaire was hosted by LimeSurvey.com, an open-source statistical web application.

Sociodemographic variables recorded for analysis included participant age, country of birth, identification with heritage group, highest educational attainment, and current employment and study status. They were also asked to report marital status, relationship satisfaction, number and age of dependent children, as well as whether they were currently pregnant.

The Alcohol Use Disorders Identification Test [AUDIT; (78)] was used to assess a participant's typical level of alcohol consumption. It consists of 10 brief items which address frequency and quantity of use, binge behavior, and indicators of dependence and harm. Only the AUDIT-Consumption (AUDIT-C) subscale was used for analysis in the current study. The AUDIT-C consists of the first three questions of the AUDIT (79), relating to frequency of alcohol consumption, the number of drinks typically consumed, and the frequency of heavy episodic drinking, defined as consuming six or more drinks on a single occasion. Responses are scored on a five-point Likert-type scale in ascending order for each question, with scores ranging from 0 to 12. According to Reinert and Allen (80), AUDIT-C scores of 3 or more indicate hazardous drinking in women. The AUDIT-C has robust psychometric properties, [Cronbach's alpha of 0.94; (81)]. It also has high concurrent validity (r = 0.97) with the full version of the AUDIT (81).

The Parenting Stress Scale [PSS; (82)] was used to measure parenting stress by capturing the demands of parenting as well as the rewards, allowing positive experiences to offset the negative. It consists of 18 self-report items, focusing on a parent's relationship with their child(ren). Level of agreement with each statement is reported on a five-point Likert-type scale. Scores range from 18 to 90, with higher scores indicating higher parental stress, and a cut-off of 54 used to define low and high stress (83). The PSS is reported to have good 6-week test-retest (0.81) reliability and internal consistency (0.83), as well as robust convergent validity against the Perceived Stress Scale (r = 0.41) and Parenting Stress Index (r = 0.75) (82). It was designed for use with parents on a wide spectrum of circum-stances (84). Compared with other scales testing the construct, the PSS has specificity in that it evaluates parenting stresses irrespective of other life stresses such as marital or financial concerns (85).

The Parenting Sense of Competence scale was created by Gibaud et al. [(86), as cited in (68)] and further developed by Johnston and Mash (68), and was used to measure self-reported competence in the parenting role. It includes 17 items with level of agreement to each statement recorded on a six-point Likert-type scale. Higher scores indicate a higher sense of competence. Parenting satisfaction, self-efficacy and interest subscales were also calculated. Rogers and Matthews (66) analyzed the scale using an Australian sample, finding alpha coefficients of 0.77 for satisfaction and 0.78 for self-efficacy when exploring mothers specifically. Six-week test-retest reliability ranges from 0.46 to 0.82 (87). The parenting sense of competence scale has also been found to have good convergent validity with measures of parental distress (88) and self-efficacy (89).

As data for this study was collected in June and July 2020, during the COVID-19 global pandemic, it is of interest to understand how this may have distorted alcohol consumption compared to typical use, and potentially confounded results. Therefore, participants were asked how their drinking has been impacted by COVID-19 (increased, decreased, or unchanged). The potential for systematic confounding effects at the time of testing was further explored by questioning participants about the influence of COVID-19 on their overall well-being.

### Procedure

Participants were recruited using a paid electronic advertisement across Facebook and Instagram. Those who clicked the link were taken to a questionnaire that they could complete after giving consent and confirming their eligibility. The questionnaire was estimated to take 30-45 min to complete, however, they could exit at any time and resume later should they wish. There was no consequence for non-completion. As the questionnaire included scales for multiple separate studies, the extent of partial responses was assessed, with data analyzed for any responder who completed the scales required in the current study. The study was approved by the Human Research Ethics Committee (H-2020-0156).

### Analysis

Descriptive statistics were used to summarize the sample characteristics of those who withdrew early and those who responded to completion, ultimately being included in analyses. Mann-Whitney *U* and Chi-Squared tests were conducted to analyze the differences between these groups to ensure that the sample under analysis was representative of the initial sample.

Pearson's Correlation was used to assess all relationships between parenting stress, alcohol consumption and overall parenting sense of competence, as well as parenting sense of competence subscales—satisfaction, self-efficacy and interest.

Moderation analysis was also conducted, whereby the effect of a third variable, the moderator, on the relationship between the predictor and outcome variable was investigated (90). We considered the moderating effect of parenting sense of competence on the relationship between parenting stress and alcohol consumption score using Hayes' (90) PROCESS software. A threshold of *p* ≤ 0.050 was used to establish statistical significance, and all variables were mean centered prior to analysis.

To further investigate the nature of this relationship, an exploratory analysis was conducted using each of the parenting sense of competence subscales as moderating variables in the relationship between parenting stress and alcohol consumption score. Hayes' (90) PROCESS software was also used for these analyses, with a statistical significance threshold of *p* ≤ 0.050 and mean centering of all variables.

Prior to moderation analyses, assumption checks were performed. Relationships were weakly linear as per visual inspection of partial regression plots and a plot of studentized residuals against the predicted values. A Durbin-Watson statistic of 1.76 confirmed independence of residuals, and visual inspection of a plot of studentized residuals versus unstandardized predicted values supported homoscedasticity. There was also no evidence of multicollinearity, as assessed by tolerance values > 0.1. While two outliers were flagged due to their studentized deleted residuals being slightly more than three standard deviations, these were deemed acceptable as they did not have leverage values > 0.2 and did not have a Cook's distance above 1. These cases were maintained in the analysis as they are true representations of participant data. Finally, the assumption of normality was met, as assessed by a histogram of standardized residuals, with a mean and standard deviation of ~0 and 1, respectively. This was further supported by visual inspection of a P-P plot of standardized residuals, and the fact that studentized residuals had respective skew and kurtosis values of <1 and 2.

A robustness analysis was also performed to ensure that the pattern of statistical significance did not change with the inclusion of covariates in the model. The covariates used were participant age, number of dependent children, and the average age of children. Only participant age had missing values (*N* = 400) which were due to human error in entering birth date. Listwise deletion was used for analyses that involved these variables.

## Results

### Sample Characteristics

Chi-squared and Mann-Whitney *U*-tests indicated that there were no significant differences between the demographic characteristics of the participants who chose to withdraw from the questionnaire compared with those who responded to completion. This indicates that there was no systematic reason for participant withdrawal, therefore, those responding to completion, and subsequently analyzed, were representative of the initial sample.

Demographic characteristics and the results of comparative analyses are detailed in Table 1.

**Table 1:** Demographic characteristics of eligible survey completers vs. non-completers.

**Variable**	**Completers** **(*N* = 406)**	**Non-completers** **(*N* = 239)**	***X*** ^**2**^	***Z***
Age (years), median (5th−95th percentile)	39.0 (29–51)	38.5 (29–52)		−1.13
**Country of birth**, ***n*** **(%)**				
Australia	326 (80.3)	180 (83.3)	0.86	
Other	80 (19.7)	36 (16.7)		
**Identification with heritage group**, ***n*** **(%)**				
Yes	51 (12.6)	28 (13.0)	0.02	
No	355 (87.4)	188 (87.0)		
**Highest educational attainment**, ***n*** **(%)**				
Postgraduate degree	132 (32.5)	67 (28.0)		−1.58
Graduate diploma/certificate	46 (11.3)	23 (9.6)		
Bachelor's degree	148 (36.5)	62 (25.9)		
Advanced diploma/diploma	27 (6.7)	23 (9.6)		
Certificate III/IV	42 (10.3)	29 (12.1)		
Year 12	9 (2.2)	8 (3.3)		
Year 11 or below (incl. Certificate I/II)	2 (.5)	4 (1.7)		
**Currently studying**, ***n*** **(%)**				
Yes	135 (33.3)	63 (29.2)	1.084	
No	271 (66.7)	153 (70.8)		
**Employment status**, ***n*** **(%)**				
Currently Employed	344 (84.7)	172 (79.6)	2.59	
Not Currently Employed	62 (15.3)	44 (20.4)		
**Marital status**, ***n*** **(%)**				
Married	293 (72.2)	156 (72.2)	0.00	
Not married	113 (27.8)	60 (27.8)		
**Relationship satisfaction**, ***n*** **(%)**				
Satisfied	314 (77.3)	155 (64.9)		
Neither satisfied nor dissatisfied	57 (14.0)	29 (12.1)		
Dissatisfied	21 (5.2)	12 (5.0)		
Not applicable	14 (3.4)	20 (8.4)		
**Children**				
Total number of children, $\bar{x}$	2.12	2.25		−1.50
Number of dependent children, $\bar{x}$	1.94	2.00		−0.52
Age of eldest child, $\bar{x}$	8.68	8.24		−0.88
Age of youngest child, $\bar{x}$	5.91	5.65		−0.51
Average age of children, $\bar{x}$	7.33	6.98		−0.76
**Currently pregnant**, ***n*** **(%)**				
Yes	10 (2.5)	6 (3.0)	0.14	
No	396 (97.5)	195 (97.0)		

*Non-completers data might include missing values due to premature exit, thus, percentages may not add to 100*.

Parenting sense of competence scores ranged from 33 to 97, with an average of 68.59 (SD = 11.86). Parenting stress scores ranged from 21 to 71, with an average of 42.76 (SD = 9.44). Details of these variables can be found in Table 2.

**Table 2:** Parenting stress and well-being of eligible survey completers.

**Variable**	***N***	***%***
Parenting stress	(*N* = 406)	
Low score (≤ 54)	360	88.7
High Score (>54)	46	11.3
Perceived effect of COVID-19 on well-being	(*N* = 406)	
Increased well-being	33	8.1
Decreased well-being	178	43.8
No change	120	29.6

The majority of participants reported drinking at least weekly (52.4%), with most drinking one or two drinks on a typical drinking day (58.4%). While 9.1% of participants were non-drinkers and 36.0% were drinkers within the guidelines, 54.9% of mothers were drinking at rates which exceed the guidelines. However, 41.4% of participants stated that they were currently drinking more than normal due to the COVID-19 pandemic. Details of these alcohol-related characteristics can be found in Table 3.

**Table 3:** Alcohol-related characteristics of eligible survey completers.

**Variable**	***n***	***%***
Frequency of alcoholic drink consumption	(*N* = 406)	
Never	37	9.1
Monthly or less	69	17.0
2-4 times per month	87	21.4
2-3 times per week	100	24.6
4 or more times per week	113	27.8
Typical number of drinks per typical drinking day	(*N* = 406)	
Non-drinker	37	9.1
1 or 2	237	58.4
3 or 4	76	18.7
5 or 6	35	8.6
7 to 9	18	4.4
10 or more	3	0.7
Frequency of consuming ≥ 6 drinks per occasion	(*N* = 406)	
Non-drinker	37	9.1
Never	164	40.4
Less than monthly	115	28.3
Monthly	45	11.1
Weekly	33	8.1
Daily or almost daily	12	3.0
Alcohol consumption	(*N* = 406)	
Non-drinker	37	9.1
Drinker within 2020 guidelines	146	36.0
Drinker exceeding 2020 guidelines	223	54.9
Perceived effect of COVID-19 on alcohol consumption	(*N* = 399)	
Increased consumption	165	41.4
Decreased consumption	49	12.3
No change	185	46.4

### Correlation Between Parenting Stress, Parenting Sense of Competence, Parenting Sense of Competence Subscales, and AUDIT-C

Descriptive statistics and Pearson's correlation coefficients were computed to assess the relationships between parenting stress, parenting sense of competence, parenting sense of competence subscales, and AUDIT-C score in adult mothers with dependent children under the age of 18 (see Table 4).

**Table 4:** Descriptive statistics and Pearson's correlation coefficients.

**Measure**	**M**	**SD**	**1**	**2**	**3**	**4**	**5**	**6**
1. P. stress	42.76	9.44	-					
2. Total PSOC	68.59	11.84	−0.76^***^	-				
PSOC Subscales:								
3. Satisfaction	18.89	4.69	−0.67^***^	0.81^***^	-			
4. Self-efficacy	28.29	5.94	−0.57^***^	0.86^***^	0.48^***^	-		
5. Interest	9.18	1.97	−0.57^***^	0.56^***^	0.39^***^	0.30^***^	-	
6. AUDIT-C	3.84	2.63	0.15^**^	−0.16^**^	−0.09	−0.12^*^	−0.17^***^	-

“*P. Stress” refers to parenting stress and “PSOC” refers to parenting sense of competence*.

* *p < 0.050*,

** *p < 0.010*,

*** *p < 0.001*.

As per visual inspection of scatterplots, all bivariate relationships were deemed to have at least weak linear associations, and no extreme outliers were present. While all variables were considered non-normal by a Shapiro-Wilk test (*p* < 0.050), visual inspection of Q-Q plots indicated only slight departures from normality, and skewness and kurtosis values were <1 for all variables. Given that correlation is somewhat robust to violations of normality (91), and that the central limit theorem would be acting on our large sample (92), this was not deemed to be a large enough violation of the assumption to warrant alternative approaches.

There was a statistically significant, strong negative correlation between parenting stress and parenting sense of competence, such that greater parenting stress was associated with lower parenting sense of competence, r = −0.76, *p* < 0.001. Parenting stress and AUDIT consumption scores also showed a statistically significant, weak positive correlation, such that AUDIT consumption increased as parenting stress increased, r = 0.15, *p* = 0.002. Likewise, there was a statistically significant, weak negative correlation between parenting sense of competence and AUDIT consumption score, such that AUDIT consumption score increased as parenting sense of competence decreased, r = −0.16, *p* = 0.002. Parenting satisfaction (r = −0.67, *p* < 0.001), self-efficacy (r = −0.57, *p* < 0.001) and interest (r = −0.57, *p* < 0.001) also had statistically significant, strong negative correlations with parenting stress such that parenting satisfaction, self-efficacy and interest all increased as parenting stress decreased. However, only self-efficacy (r = −0.12, *p* = 0.013) and interest r = −0.17, *p* < 0.001) were significantly correlated with AUDIT consumption score.

### Moderation of the Association Between Participants' Parenting Stress and Alcohol Consumption

**Figure 2:**
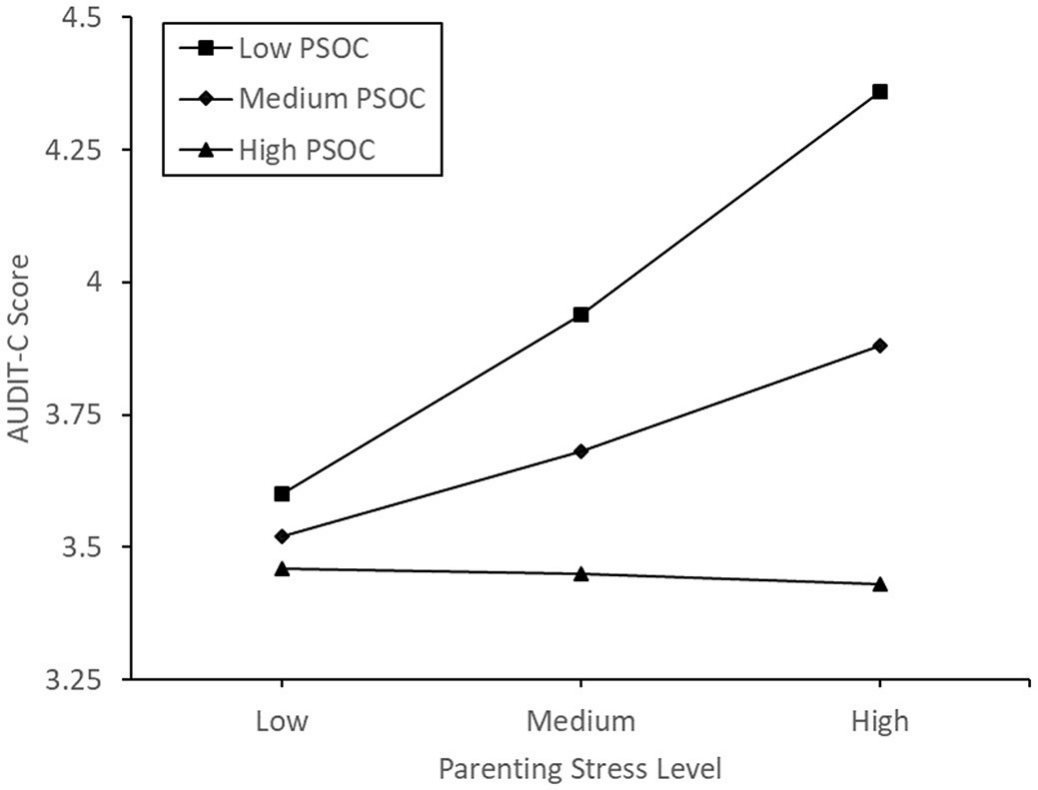
Conditional effects of parenting sense of confidence on the correlation between parenting stress and alcohol consumption.

Model 1 in Hayes' (90) PROCESS software was used to examine parenting sense of competence as a potential moderator of the association between participants' parenting stress and alcohol consumption score. There was no significant effect of parenting stress (*p* = 0.382), parenting sense of competence (*p* = 0.212), nor an interaction effect of parenting stress and parenting sense of competence (*p* = 0.136) on alcohol consumption (For further details, please see Supplementary Output A). A plot of conditional effects can be seen in Figure 2. When including the overall parenting sense of competence as the moderating variable, controlling for the covariates did not lead to a change in the pattern of significance.

### Analysis of Parenting Sense of Competence Subscales as Moderators of the Association Between Participants' Parenting Stress and Alcohol Consumption Score

**Figure 3:**
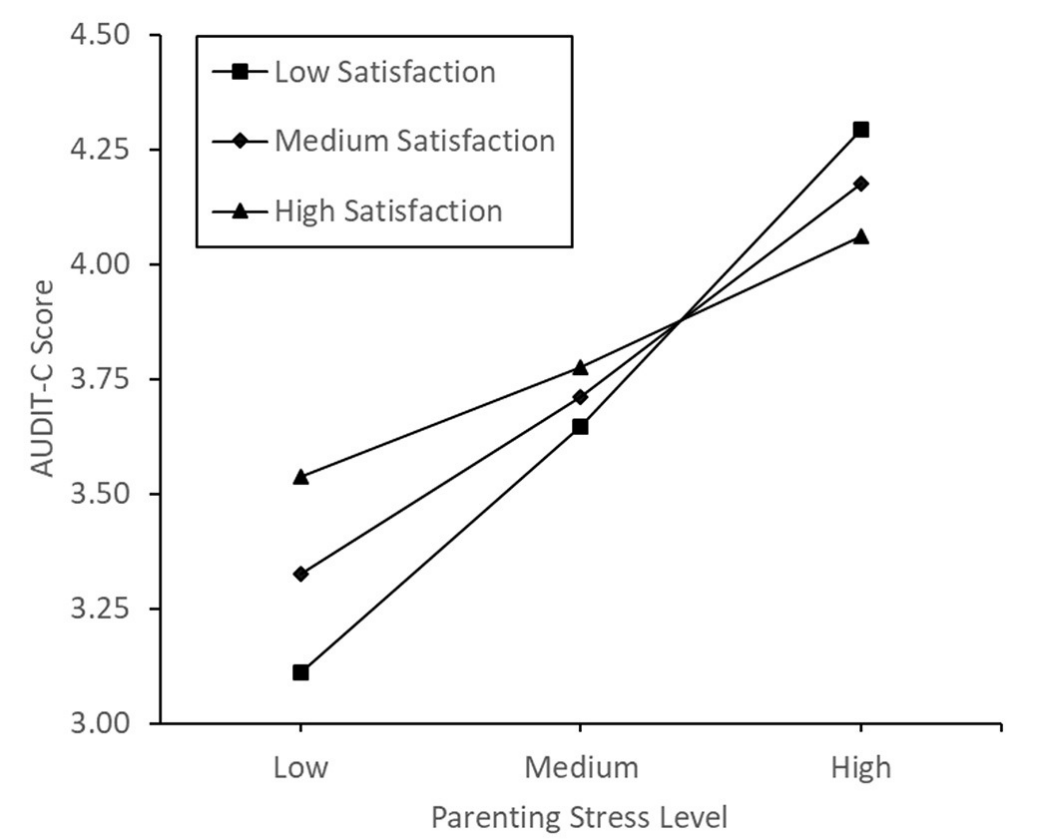
Conditional effects of parenting satisfaction on the correlation between parenting stress and alcohol consumption.

Model 1 in Hayes' (90) PROCESS software was used to assess the satisfaction sub-scale of parenting sense of competence as the potential moderator of the association between participants' parenting stress and alcohol consumption score. There was a significant main effect of parenting stress, b = 0.04, SE = 0.02, *t*_(402)_ = 2.32, *p* = 0.021, 95% CI [0.01, 0.08]. However, there was no significant effect of satisfaction (*p* = 0.784), nor an interaction effect of PSS and satisfaction (*p* = 0.257) on alcohol consumption (for further details, please see Supplementary Output B). A plot of conditional effects can be seen in Figure 3. When including the parenting satisfaction subscale as the moderating variable, controlling for the covariates did not lead to a change in the pattern of significance.

**Figure 4:**
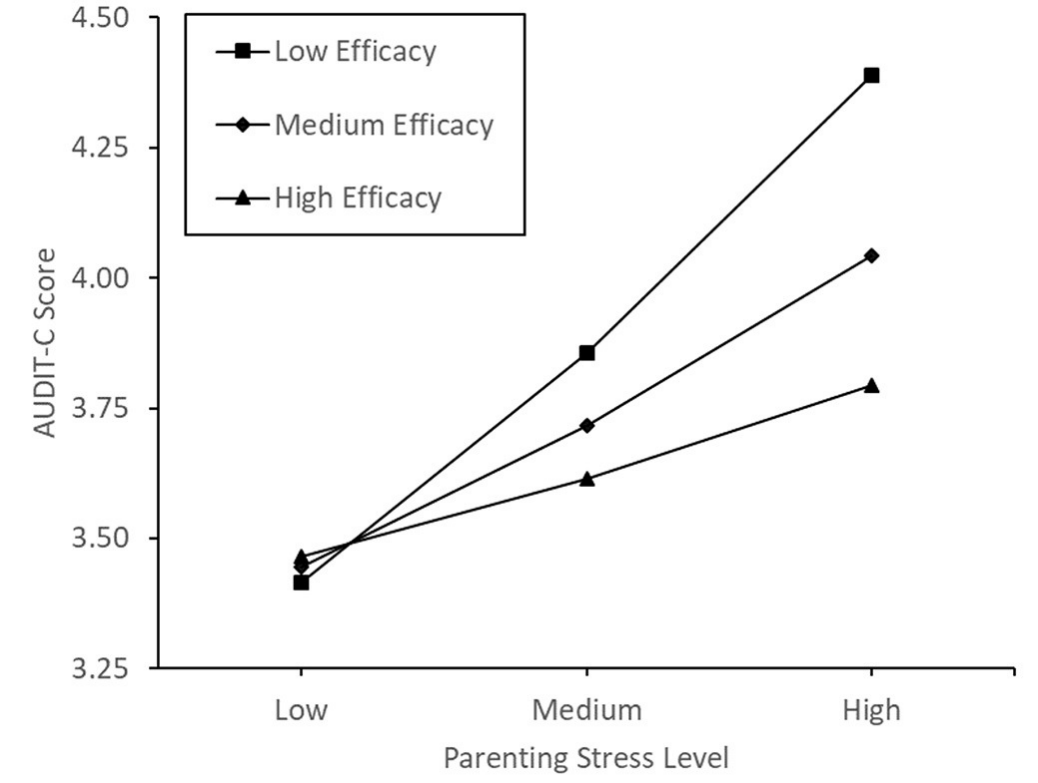
Conditional effects of parenting self-efficacy on the correlation between parenting stress and alcohol consumption.

Model 1 in Hayes' (90) PROCESS software was used to assess the self-efficacy sub-scale of parenting sense of competence as the potential moderator of the association between participants' parenting stress and alcohol consumption score. There was no significant main effect of parenting stress (*p* = 0.056) or self-efficacy (*p* = 0.403), nor an interaction effect of parenting stress and self-efficacy (*p* = 0.207) on alcohol consumption (for further details, see Supplementary Output C). See Figure 4 for a plot of conditional effects. When testing parenting self-efficacy as the moderator, inclusion of the covariates forced the main effect of parenting stress into significance, b = 0.04, SE = 0.02, *t*_(396)_ = 2.24, *p* = 0.026, 95% CI [0.00, 0.07], with no change elsewhere.

Model 1 in Hayes' (90) PROCESS software was used to assess the interest subscale of parenting sense of competence as the potential moderator of the association between participants' parenting stress and alcohol consumption score. There was no significant main effect of parenting stress (*p* = 0.250) or interest (*p* = 0.108), however, there was a significant interaction effect of parenting stress and interest on alcohol consumption, b = −0.01, SE = 0.01, *t*_(402)_ = −2.08, *p* = 0.038, 95% CI [−0.03, −0.00].

**Figure 5:**
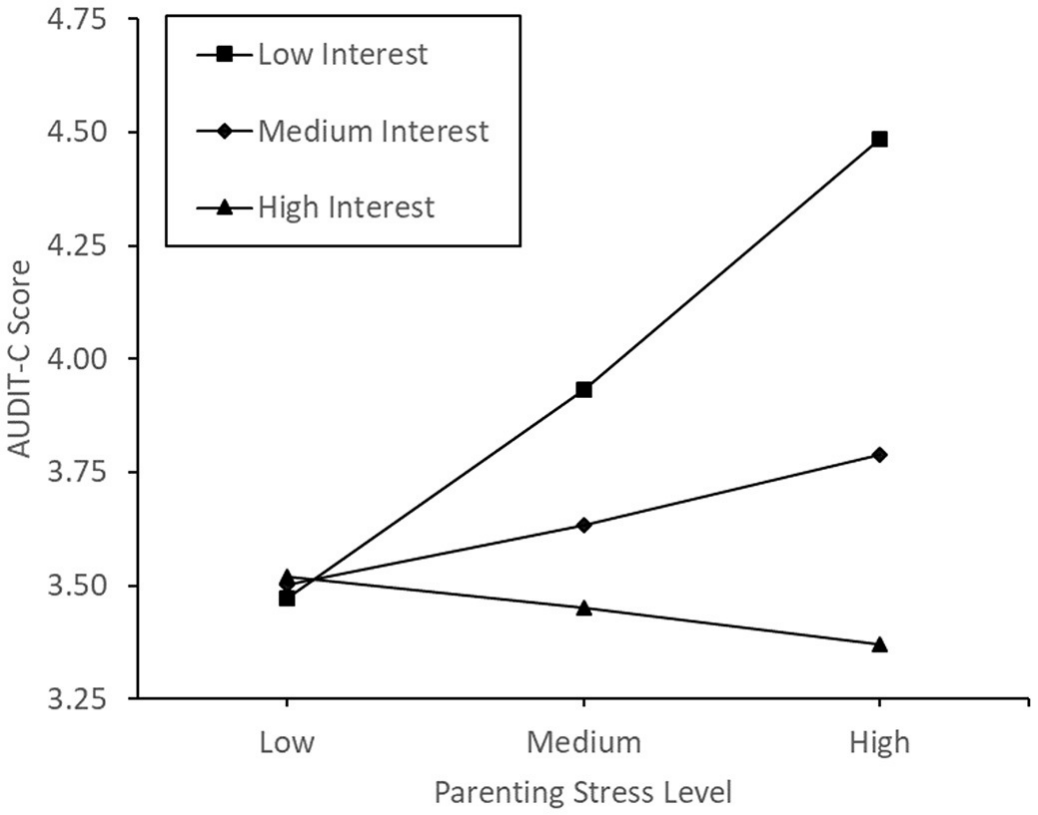
Conditional effects of parenting interest on the correlation between parenting stress and alcohol consumption.

To investigate this interaction, the conditional effects of parenting stress on alcohol consumption at low, medium and high levels of parenting interest were examined. These levels were defined by the 16th, 50th, and 84th percentiles of the mean centered data. The effect of parenting stress on alcohol consumption was only significant at the low level of parenting interest, b = 0.05, SE = 0.02, *t*_(402)_ = 2.36, *p* = 0.019, 95% CI [0.01, 0.09], but not at the medium (*p* = 0.396) or high levels (*p* = 0.731) (see Figure 5).

Controlling for covariates with parenting interest as the moderator, forced the main effect of parenting interest into significance, b = −0.17, SE = 0.08, *t*_(396)_ = −2.07, *p* = 0.039, 95% CI [−0.33, −0.01], however, the previously significant interaction effect of parenting stress and interest on alcohol consumption (*p* = 0.038) became insignificant (*p* = 0.078). Therefore, the moderation of parenting interest on the relationship between parenting stress and alcohol consumption should be viewed with caution.

### Robustness Analysis

As part of a robustness check, moderation analyses were repeated, adding relevant covariates to establish that results were not dependent on their inclusion or exclusion. Covariates included were participant age, number of dependent children aged under 18, and the average age of children.

## Discussion

The current study aimed to assess the correlations between parenting stress and alcohol consumption in a non-clinical sample of Australian mothers, while investigating potential moderation by parenting sense of competence. As hypothesized, parenting stress was positively correlated with alcohol consumption, suggesting that as a mother's parental stress rises, her alcohol consumption tends to increase. This pattern of drinking is consistent with a wide body of literature finding a positive relationship between stress and alcohol use (12, 34, 63, 64), but is also inconsistent with those studies suggesting the opposite (12, 13). This discrepancy might be explained by differing methodologies, with Pelham et al., utilizing an experimental design in which parents were stressed and then offered alcohol whilst Breslin et al., collected self-report data on levels of parenting stress and alcohol consumption. The influence of respondents wishing to minimize their alcohol use may have resulted in self-report data underestimating alcohol consumption in the Breslin et al., study, however if this were the case it would also be expected in the current study where results consistent with Pelham et al., were observed.

The results provided support for the hypothesis that parenting stress is negatively correlated with parenting sense of competence, indicating that as a mother's parenting stress increases, she is less likely to view her parenting ability favorably, exhibiting lower confidence in her role as a parent. Consistent with predictions, there was also a significant, weak negative association between alcohol consumption and parenting sense of competence. This indicates that as a mother's perception of her parenting competence improves, her alcohol consumption tends to decrease. However, there was no significant moderation effect of parenting sense of competence on the relationship between parenting stress and alcohol consumption, therefore, our final hypothesis was not supported.

These results suggest that highly stressed mothers are a group particularly vulnerable to alcohol misuse. Given the continuous monitoring of child development, there are several opportunities for the informal assessment of mothers by health professionals. This would allow for intervention where needed, without risk of the judgement and stigma often associated with specifically alcohol-related help-seeking of mothers (93–95). Alcohol misuse should be screened for, detected and treated simultaneously with the management of parenting stress, along with the building of a strong sense of competence in the parental role. This is especially pertinent for mothers using alcohol to cope with stress, as it may facilitate the use of more adaptive strategies.

We hypothesized that parenting sense of competence would reduce or reverse the correlation between parenting stress and alcohol consumption. Contrary to predictions, parenting sense of competence was not a significant moderator of the relationship between parenting stress and alcohol consumption. Despite this, conditional plots of the data did portray the expected pattern of influence, with the positive correlation between parenting stress and alcohol use weakening as parenting sense of competence increases, eventually becoming negative. Following this result, we explored the construct more deeply, performing separate moderation analyses on the subscales. The satisfaction and self-efficacy subscales of parenting sense of competence were not significant moderators of the relationship between parenting stress and alcohol consumption. Conversely, the interest subscale of parenting sense of competence was a significant moderator of the relationship between parenting stress and alcohol consumption, with those holding a low interest in parenting being more likely to consume alcohol during high parenting stress. However, when controlling for maternal age, the number of dependent children and average age of children, this moderation was no longer significant, and thus, should be viewed with caution.

A possible explanation for the non-significant moderation of parenting sense of competence is that this data was collected during the first 6-months of the COVID-19 pandemic. Studies have shown that middle-aged Australian women were consuming more alcohol than they typically do during this time (34, 96, 97), stating that they were doing so to cope with a range of factors, especially their extended time at home and increased stress (34). Participants in the current study reported the same effect, with over a third reporting that their drinking had increased as a result of COVID-19. While the current sample had maternal parenting sense of competence comparable to a similar study by Studts et al. (98), it was a relatively low-stress group. The proportion of those categorized as experiencing high parenting stress in the current study was approximately half the share found in a similar study by Rajgariah et al. (83). The combination of analyzing a particularly low stressed group, during a time distinguished by amplified drinking, could explain the weaker than expected correlation between parenting stress and alcohol consumption. The weak correlation may rationalize the non-significant moderation of parenting sense of competence, despite plots indicating an interaction. Therefore, future research should attempt to replicate the moderation analysis during a time less confounded by systematic social and emotional disturbance.

This study has several limitations. First, the results may be subject to response bias, whereby the individuals who self-nominated to complete the questionnaire were systematically different from those who did not (99). While we did not find significant differences in the demographics of those who withdrew before completion and those who completed, we cannot compare responders with those who did not begin the questionnaire at all. Secondly, results rely on self-reported data. Whilst we attempted to minimize socially desirable responding via full anonymity, participant error in interpreting and answering questions, particularly around the number of standard drinks consumed, could not be measured or controlled for. To mitigate this risk, participants were provided with images representing the standard drink sizes of commonly consumed types of alcohol, however, drinkers often underestimate the amounts they consume, responding with intrinsic bias (100). Additionally, it is possible that the 37% of eligible participants who withdrew could have dramatically changed the results. This is especially concerning given that they place the current study well below the ideal response rate for reducing response bias of 85% (99). One likely reason for the high drop-out rate is the length of the questionnaire (101), which included additional research outside of the current study. This was likely to introduce a fatigue effect, not only causing participants to withdraw, but potentially confounding responses of those who carried on as they begin to use shortcuts such as acquiescence or fence-sitting (102). Finally, the observational nature of this study only allows us to gather descriptive data, but no claims about causality or temporal precedence can be made.

While this study did not find parenting sense of competence to be a significant moderator on the relationship between parenting stress and alcohol use, it was the first study to establish a direct negative correlation between parenting sense of competence and alcohol consumption. Given the already established inverse relationship between parenting sense of competence and parenting stress, there appears to be strong potential for the manipulation of parenting sense of competence in treating alcohol-related problems and parenting stress simultaneously.

**Figure 6:**
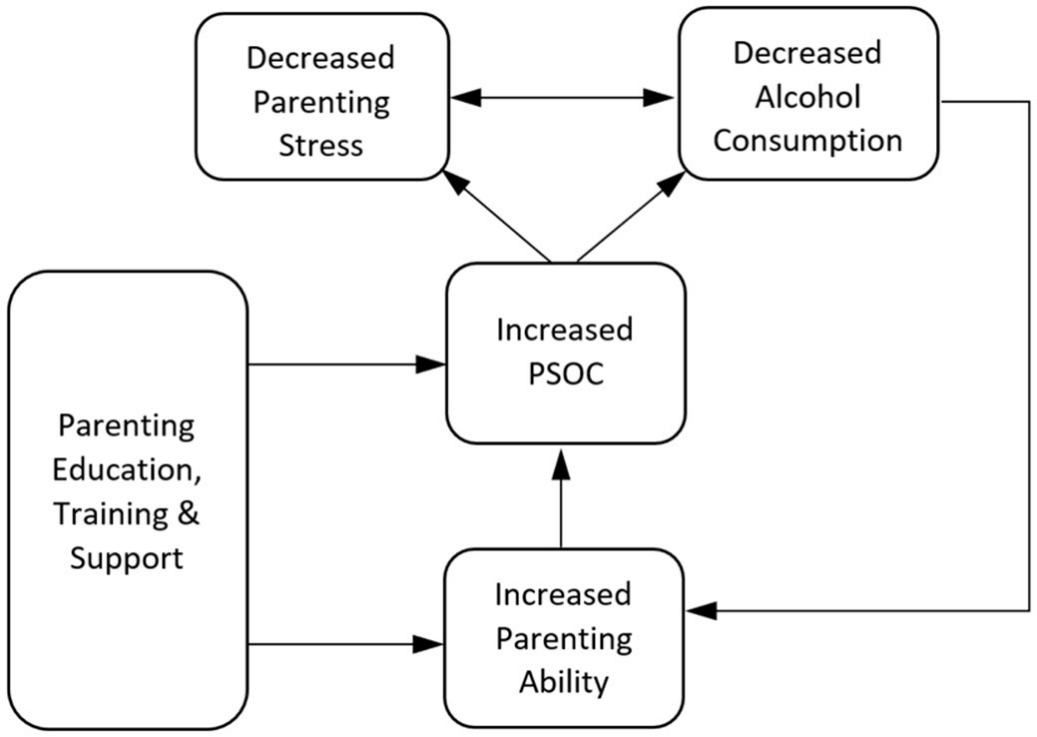
Cycle of parenting sense of competence, parenting stress and alcohol consumption with parenting intervention.

Feldman and Werner (103) indicated that long-term changes in parenting sense of competence can be gained via treatments targeting parenting ability. This can be done both formally and informally, with differing levels of coercion and choice involved, ranging from formal interventions such as Project SafeCare (104), to less formal means of improving parenting ability including house visits, parenting groups and social interaction (105). A meta-analysis by Barlow et al. (106) showed that treatments designed to improve parenting ability also contribute to improvements in psychological distress. Thus, it is reasonable to assume that such improvements could help reduce the incidence of coping-motivated alcohol use when faced with high parenting stress. These interventions could break the negative self-propagating cycle of stress and alcohol consumption, triggering a new cycle of more effective parenting, increased confidence, less stress and reduced alcohol consumption (Figure 6). Furthermore, parenting sense of competence has been found to significantly predict both informal and formal alcohol-related help-seeking (107), so improvements act both directly, but also indirectly, as parents become more likely to seek treatment for other risk factors.

The finding that parenting sense of competence is related to alcohol misuse may prompt the acknowledgment of these informal strategies as key in preventing and treating alcohol misuse. This recognition may allow for execution of these strategies at a wide community level, reaching a broader collection of people than formal, alcohol-related help-seeking.

Overall, our findings attempt to explain an inconsistency in the literature which suggests that some women consume more alcohol when parenting stress is high, while others drink less. We supported the findings of previous research that parenting stress is positively associated with alcohol consumption (12, 34, 63, 64) and negatively correlated with parenting sense of competence (36, 71, 72). We also found parenting sense of competence to be negatively related to maternal alcohol use, a finding which was previously not explored in psychological literature. However, under the conditions of this study, the association between parenting stress and alcohol consumption was not as strong as expected, rendering the moderation by parenting sense of competence non-significant. Despite the limitations of the current study, parenting sense of competence appears to be a promising opportunity for future prevention and intervention in maternal alcohol misuse, particularly that motivated by coping.

Future research should aim to replicate the current study at a time with less social, financial and parental turbulence. A shorter, more focused questionnaire is also recommended; however, the addition of experimental methodology would facilitate more causal inference and reduce self-report bias. Given that this is the first study to explore the moderating effect of parenting sense of competence on parenting stress and alcohol, as well as the first connecting parenting sense of competence to alcohol directly, expansion of this concept may be triggered in future research. With further exploration, this concept has the potential to aid in the advancement of targeted prevention and intervention, improving alcohol-related care, and supporting parents in need.

## Data Availability Statement

The original contributions presented in the study are included in the article/Supplementary Material, further inquiries can be directed to the corresponding author.

## Ethics Statement

The studies involving human participants were reviewed and approved by the University of Newcastle Human Research Ethics Committee. The patients/participants provided their written informed consent to participate in this study.

## Author Contributions

EJ contributed to project design and data collection, carried out all analyses, and wrote the first draft of this manuscript. RF and KC contributed to project design and data collection. SH initiated the project design, contributed to data collection, oversaw all steps in this project, and edited the final draft of the manuscript. All authors contributed to the article and approved the submitted version.

## Funding

This research was conducted by an employee and students of the University of Newcastle and was not externally funded.

## Conflict of Interest

The authors declare that the research was conducted in the absence of any commercial or financial relationships that could be construed as a potential conflict of interest.

## Publisher's Note

All claims expressed in this article are solely those of the authors and do not necessarily represent those of their affiliated organizations, or those of the publisher, the editors and the reviewers. Any product that may be evaluated in this article, or claim that may be made by its manufacturer, is not guaranteed or endorsed by the publisher.
