# Supplementary material for: Self-Assured and Sober: The Relationship Between Maternal Parenting Sense of Competence, Stress, and Alcohol Use
Source: Front Glob Womens Health. 2022 Jan 31;2:778183. doi: 10.3389/fgwh.2021.778183 (PMC8841786; doi:10.3389/fgwh.2021.778183)
Supplement: Supplementary file 1 [file Data_Sheet_1.PDF]

Supplementary Analysis Output

Supplementary Output A

Significance Values for Main and Interaction Effects on AUDIT-C Score – Total PSOC

Measure	*b*	*SE*	*t*	*p*	*LLCI*	*ULCI*
Parenting Stress	0.02	0.02	0.88	.382	-0.02	0.06
PSOC	-0.02	0.02	-1.25	.212	-0.05	0.01
PSS x PSOC	-0.00	0.00	-1.49	.136	-0.00	0.00

Note. * $p < .050$, ** $p < .010$, *** $p < .001$

Supplementary Output B

Significance Values for Main and Interaction Effects on AUDIT-C Score – Satisfaction

Measure	*b*	*SE*	*t*	*p*	*LLCI*	*ULCI*
Parenting Stress	0.04	0.02	2.32	.021*	0.01	0.08
Satisfaction	0.01	0.04	0.27	.784	-0.06	0.08
PSS x Satisfaction	-0.00	0.00	-1.13	.257	-0.01	0.00

Note. * $p < .050$, ** $p < .010$, *** $p < .001$

Supplementary Output C

Significance Values for Main and Interaction Effects on AUDIT-C Score – Self-Efficacy

Measure	*b*	*SE*	*t*	*p*	*LLCI*	*ULCI*
Parenting Stress	0.03	0.02	1.91	.056	-0.00	0.06
Self-Efficacy	-0.02	0.03	1.84	.403	-0.07	0.03
PSS x Self-Efficacy	-0.00	0.00	-1.26	.207	-0.01	0.00

Note. * $p < .050$, ** $p < .010$, *** $p < .001$
